# Rare phenotypes of white coat color in Simmental calves: genetic causes of syndromic forms of albinism and depigmentation

**DOI:** 10.1007/s00438-025-02290-2

**Published:** 2025-09-06

**Authors:** Joana G. P. Jacinto, Therese Leuenberger, Miriam Hauser, Irene M. Häfliger, Franz R. Seefried, Anna Letko, Cord Drögemüller

**Affiliations:** 1 Clinic for Ruminants, Department of Clinical Veterinary Medicine, Vetsuisse Faculty, University of Bern, 3012 Bern, Switzerland; 2 Institute of Genetics, Vetsuisse Faculty, University of Bern, 3012 Bern, Switzerland; 3 Qualitas AG, Zug, Switzerland

**Keywords:** Bovine, Precision medicine, Skin, Pigmentation, Leucism

## Abstract

The aim of this study was to investigate three unrelated Simmental calves with atypical white coat color, identify potential genetic causes using a trio-based whole-genome sequencing approach, and assess the prevalence of the identified variants in the breed. Several inherited alleles affecting coat color, ranging from fawn to red spotted and white-headed, have been described in Simmental cattle originating from Switzerland. However, no genetic variant has yet been associated with an almost completely white coat in this breed. Clinical examination revealed different syndromic disorders of white coat color in Simmental in all three cases, and pedigree records indicated recessive inheritance. Filtering for rare protein-changing variants revealed an independent homozygous variant that could be the cause in each case: a likely pathogenic missense variant in *TYR* (NP_851344.1:p.Pro428Leu) in case 1 with oculocutaneous albinism type 1, a likely pathogenic missense variant in *GRID1* (XP_024842694.1:p.Pro489His) in case 2 with short stature-auditory depigmentation syndrome, and a frameshift variant of uncertain significance in *RAD54B* (NP_001179884.1:p.Ala722_Gly724delinsAsnLeuIlePheCys*) in case 3 with a multisystem depigmentation syndrome. Validation by Sanger sequencing confirmed the variant genotypes, and parental heterozygosity supported recessive inheritance. These variants were almost entirely absent from other breeds, and the allele frequency of the three candidate causal variants was less than 1% in the current Swiss Simmental population. This study identified three novel recessive alleles associated with syndromic forms of albinism or depigmentation, revealing unexpected heterogeneity. The investigation did not reveal any indications of possible dominant de novo mutations impacting protein coding genes including known candidate genes for depigmentation phenotypes. These findings possibly expand the list of pigmentation related genes in mammals, but further investigation is needed. We also highlight the biomedical relevance of investigating rare congenital disorders in livestock.

**Supplementary Information:**

The online version contains supplementary material available at 10.1007/s00438-025-02290-2.

## Introduction

The aim of this study was to characterize the clinical phenotypes of a series of three unrelated Simmental calves reported to be born breed-atypically white to normal parents, to search for possible causative genetic variants using a whole-genome sequencing (WGS) trio approach and to estimate their prevalence in the Simmental population of Switzerland.The genetic and phenotypic diversity of *Bos taurus* has been influenced by natural selection, domestication, and breeding selection (Gutiérrez-Gil et al. 2015). Worldwide, there are hundreds of cattle breeds, and characteristics such as coat color, spotting, and depigmentation patterns are prominent and easily observable traits, playing important roles in breed identity, selection, and in some cases, animal health (Cieslak et al. 2011). Understanding the genetic basis of coat color variation has not only influenced breeding practices but has also provided insights into pigmentation biology relevant across mammalian species (Cieslak et al. 2011). While many coat color phenotypes in cattle are well-characterized, rare congenital pigmentation anomalies, such as extensive depigmentation or albinism, remain unexplored.

Studies of the genetic basis of coat color variation in cattle have identified several genes like *MC1R*, *KIT*, and *ASIP* with significant effects, some of which also influence other traits (Joerg et al. 1996; Durkin et al. 2012; Trigo et al. 2021; He et al. 2022). These coat color variations are due to naturally occurring mutations, and the derived alleles are often spread through selective breeding, as the distinctive appearance of these animals is highly prized and helps to define the breeds themselves.

Depigmentation (‘leucism’), a lack of mature melanocytes in the unpigmented (‘white’) areas of the skin, in cattle can range from the common white markings, which can occur in a variety of patterns, from small white spots or patches on the head and legs to large irregular white patches, large symmetrical patterns such as white bands around the body, or marked depigmentation to completely white (Olson 1981). In cattle, variants associated with *TYR*,* SLC45A2*, and *KIT*, are related with different white coat color patterns, while variants in *TYRP1*, *FZD7*,* MITF*, *TWIST2* and *PMEL* can result in visible, sometimes ‘almost-white’ coat color dilution (Schmutz et al. 2004; Kühn and Weikard 2007; Philipp et al. 2011; Awasthi Mishra et al. 2017; Häfliger et al. 2020a; Bhati et al. 2020; Floriot et al. 2021; Petersen et al. 2023; Wang et al. 2023; Milia et al. 2024). More rarely, inherited forms of albinism, characterized by a complete absence of melanin, particularly in the eyes, skin and hair, are also known in cattle. In particular, a recessively inherited *TYR*-related form of the coat/skin color, oculocutaneous albinism type I (OMIA:000202–9913), and a *SLC45A2*-related form of coat color, albinism, oculocutaneous type IV (OMIA:001821–9913) have been reported in Brown Swiss (Schmutz et al. 2004; Rothammer et al. 2017; Bhati et al. 2020).

The light yellowish tan (‘fawn’) to dark red spotted and white-headed Simmental cattle originating from Switzerland are all homozygous for a recessive loss-of-function allele in *MC1R*, designated *e* (OMIA 001199–9913, Variant ID 485), resulting in the sole production of pheomelanin pigment, whereas the variation in coat color dilution is explained by a semidominant acting coding allele in *PMEL* (OMIA:001545–9913, Variant ID 484), and the characteristic dominant inherited white head pattern is associated with a segmental duplication upstream of *KIT* (Milia et al. 2024) (OMIA:001737–9913, Variant ID 1764). However, to date, no variants have been associated with depigmentation resulting in a completely or almost completely white coat in Simmental cattle.

## Methods

### Animals and clinical investigation

This study did not require official or institutional ethical approval as it was not experimental, but rather part of routine clinical veterinary diagnostics. All animals in this study were examined with the consent of their owners and handled according to good ethical standards. Three purebred Simmental calves (cases 1–3) born with an atypical amount of white coat color to normal parents were reported to the Institute of Genetics, University of Bern (Online Resource 1). The cases originated from three different farms. In addition to the white coat color, case 1 was presented because of ocular light sensitivity, case 2 because of short stature, and case 3 because of enlarged navel. The three unrelated calves underwent a thorough clinical examination at the farm.

### DNA extractions

Genomic DNA was extracted from samples of the three affected animals (EDTA blood), three dams (EDTA blood), and three sires (semen) using Promega Maxwell RSC DNA system (Promega, Dübendorf, Switzerland).

### Whole-genome sequencing and variant calling

WGS data was generated using the Illumina NovaSeq6000 (Illumina Inc., San Diego, CA, USA) on the genomic DNA extracted from samples of the three cases and their parents. The sequenced reads were mapped to the ARS-UCD1.2 reference genome (Rosen et al. 2020), resulting in an average read depth of 15.8×, and single-nucleotide variants (SNV) and small indel variants were called. The applied software and steps to process fastq files into binary alignment map and genomic variant call format (VCF) files were in accordance with the 1000 Bull Genomes Project processing guidelines of run 7 (Hayes and Daetwyler 2019), except for the trimming, which was performed using fastp (Chen et al. 2018). Downstream processing of the genomic data was performed as reported previously (Häfliger et al. 2020b). The effects of all called variants were functionally evaluated with snpEff v5.0c (Cingolani et al. 2012), using the NCBI Annotation Release 106 (https://www.ncbi.nlm.nih.gov/genome/annotation_euk/Bos_taurus/106/). This resulted in the final VCF file, comprising jointly genotyped individual variants and their functional annotations.

To identify variants associated with the phenotype, the genotypes of the trios were compared to 1035 cattle genomes of different breeds sequenced as part of the ongoing Swiss Comparative Bovine Resequencing project, including 101 other purebred Simmental cattle. All WGS data are available in the European Nucleotide Archive (project accession numbers PRJEB18113 and PRJEB83441; Online Resource 1). Regarding possible autosomal recessive inheritance, two different scenarios were hypothesized for variant filtering: (1) a homozygous allele common to all cases and (2) independent private alleles considered individually in each case. Additionally, regarding possible dominant de novo mutations, two different scenarios were hypothesized for variant filtering: (1) a heterozygous allele, considering each case as an isolated post-zygotic event, and (2) a heterozygous allele, inherited from the sire’s mosaic germline, considering each case individually.

### Runs of homozygosity analysis

PLINK v1.9 (Chang et al. 2015) was used for quality control pruning of the initial 54,305,526 variants called in all 110 purebred Simmental genomes extracted from the Swiss Comparative Bovine Resequencing project. Only high-quality biallelic SNVs mapped to the 29 bovine autosomes called in all individuals with minor allele frequency > 0.05 and Hardy-Weinberg equilibrium exact test p-value > 1 × 10^−6^ were retained for the downstream analyses, which constituted a set of 11,650,091 SNVs. Parentage within the three trios was verified with the estimated relatedness for each pair of samples using the identity-by-descent calculation. Genome-wide search for runs of homozygosity (ROH) in each individual calf as well as regions shared by the three calves was performed using the R package detectRUNS v.0.9.6 (Biscarini et al. 2019). Based on published guidelines (Gorssen et al. 2021), the following parameters were set for the ROH detection: sliding window size of minimum 20 SNVs, maximum of 4 heterozygous genotypes in a window, minimum ROH length of 200 kb, minimum number of 66 SNVs in a ROH, and minimum density of one SNV per 50 kb. Additionally, homozygosity-based genomic inbreeding coefficient (F_ROH) was calculated based on the total length of the autosomal genome covered by SNV positions (herein 2,488,313,529 bp) and the F_ROH within the studied trios were compared to the mean F_ROH obtained from the analysis of 101 control Simmental genomes.

### In silico assessment of the molecular consequences

PredictSNP1 (Bendl et al. 2014), which includes PolyPhen-1, Polyphen-2 (Adzhubei et al. 2013), SIFT (Ng and Henikoff 2003), and PhD-SNPg (Capriotti and Fariselli 2023), and MutPred2 (Pejaver et al. 2017) were used to predict the biological consequences of the candidate variants.

### Occurrence of variants in a global control cohort

The comprehensive variant catalogue from run 9 of the 1000 Bull Genomes Project was available to investigate the allelic distribution of variants within a global control cohort (Hayes, and Daetwyler, 2019). The full dataset includes 5116 bovine genomes, including 576 from the Swiss Comparative Bovine Resequencing Project, from a wide variety of more than 130 breeds.

In addition, eight of the identified protein-changing variants in *FASN*, *GRID1*,* GRN2A*,* MAGI1*, *NSMF*,* OTOGL*,* TYR*, and *ZNF226*, were added to the subsequently updated versions of the Swiss Axiom custom genotyping arrays (Thermo Fisher Scientific, Waltham, MA, USA) routinely used for genomic selection in Switzerland. Thus, after some time of population-wide genotyping in Swiss dairy cattle for the purpose of genomic selection, several hundreds to thousands of genotypes for these variants were available, mainly in the five largest breeds (Brown Swiss, Original Braunvieh, Holstein, Swiss Fleckvieh, and Simmental). Evaluation of the prevalence of these deleterious alleles in Simmental and the four other populations was performed.

### Candidate gene and candidate variant classification

The term ‘candidate gene’ was used to describe the genes based on function and/or associated white coat/hair-related phenotypes in mammalian species. Known candidate genes for depigmentation phenotypes in cattle include *DCT* (OMIM:619165), *EDNRA* (OMIA:002164–9925), *EDN3* (OMIM:131242), *KIT* (OMIA:000209–9913), *KITLG* (OMIA:000213–9863) *LRMDA* (OMIM:615179), *MFSD12* (OMIA:002197–9615), *MITF* (OMIA:001680–9913), *OCA2* (OMIM:611409), *OCA5* (OMIM:615312), *PAX3* (OMIA:000214–9913), *PMEL* (OMIA:001545–9913), *SLC24A5* (OMIA:002124–9796), *SLC45A2* (OMIA:001821–9913), *SOX10* (OMIA:002453–9823), *TRPM1* (OMIA:002139–9796), *TYR* (OMIA:000202–9913), and *TYRP1* (OMIM:203290) (Thomas and Erickson 2008; Fleck et al. 2016).

The term ‘candidate variant’ was used to describe variants that took into account the affected gene function and/or associated phenotype in mammalian species, rarity, and the predicted effect of the variant on the encoded protein, with at least three in silico tools predicting it to be deleterious. Candidate variants were considered for detailed analysis if they occurred no more than once in the homozygous state across the entire cohort of control genomes when a recessive mode of inheritance was hypothesized. Furthermore, candidate variants were considered for detailed analysis if they were absent in the heterozygous state across the entire cohort of control genomes excluding the case’s sire, whose semen DNA was utilized for sequencing, when a dominant acting *de novo* mutation was hypothesized. In addition, the identified protein-changing variants were further classified according to the standards and guidelines for the interpretation of sequence variants in human and animal genetics (Richards et al. 2015; Boeykens et al. 2024). All sequence accessions used for the candidate variants are listed in Online Resource 1.

IGV software version 2.0 (Robinson et al. 2017) was used for visual assessment of genomic regions containing the functional candidate genes, as well as for visual inspection of candidate variants resulting from variant filtering.

### Targeted genotyping

PCR and Sanger sequencing were used to validate and genotype candidate variants. PCR products from genomic DNA were amplified using AmpliTaq Gold 360 Master Mix (Thermo Fisher Scientific, Waltham, MA, USA) and the PCR amplicons were directly sequenced on an ABI3730 capillary sequencer (Thermo Fisher Scientific, Darmstadt, Germany). The primer details are listed in Online Resource 2.

## Results

### Clinical phenotype

**Fig. 1:**
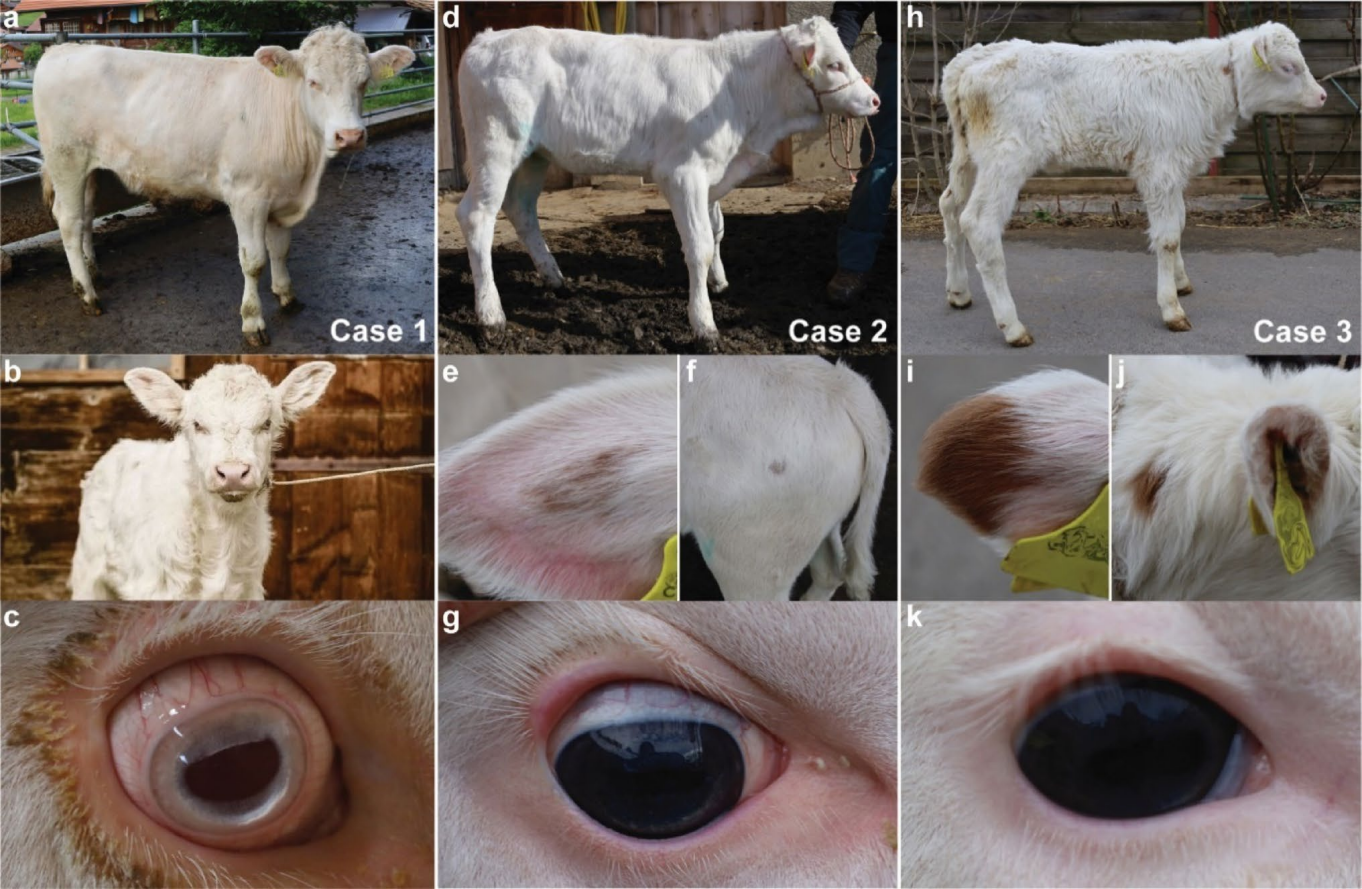
Phenotypic features of three Simmental calves born with white coat color. Case 1 **a-c** at **a** 15 months and **b** two months with oculocutaneous albinism. Note that the calf has no pigmentation. **c** Light blue irides, congestion of episcleral vessels, and purulent eye discharge visible in the periocular region. Case 2 **d-g** at four months with an almost completely depigmented coat and proportionally short stature. **e-f** Note the two red pigmented areas in the left ear and left greater femoral trochanter. **g** The iris is pigmented. Case 3 **h-k** at one week old with nearly completely depigmented coat and a reduced nutritional status. **i** Note the two red pigmented areas at the tip of the left ear and between the ears. **k** The iris and sclera are pigmented

The three Simmental calves were born with an atypical, almost white coat color (Fig. 1). All parents showed the typical red spotted and white-headed coat color pattern of purebred Simmental cattle. No unusual depigmentation phenotypes were reported in any of the available ancestors and relatives.

 Case 1 was a two-month-old male Simmental calf with a complete white coat (Fig. 1**a**-**c**). On clinical examination, the calf had a pale pink oculoconjuctival mucosa, light blue irises, congested episcleral vessels, purulent ocular discharge and unpigmented cilia bilaterally. The muzzle was also depigmented. The calf presented with physiological visual acuity, however when exposed to daylight, the animal presented semi-closed eyes, indicating photosensitivity. Claws were partially pigmented. No other abnormalities were noted on clinical examination. According to the owner, the animal was in good general condition at the age of 15 months, except for the ocular photosensitivity. The clinical findings in case 1 were consistent with a form of oculocutaneous albinism (OCA).

Case 2 was a four-month-old female Simmental calf (Fig. 1**d**-**g**). The animal had a proportionally short stature and a predominantly white coat color with two subtle red pigmented spots. One was triangular and located on the left ear pinna; the second one was round and approximately two centimeters in diameter located in the region of the left greater femoral trochanter. The oculoconjuctival mucosa were pale pink, the muzzle was pink, the iris and sclera were pigmented, and the cilia were depigmented. The calf displayed a mild brachygnathia. In addition, a reduced response to auditory stimuli was noticed. According to the owner, the animal remained very small and never showed signs of heat until the age of 30 months and was therefore slaughtered. The clinical findings in case 2 were consistent with a diagnosis of short stature-auditory depigmentation syndrome.

Case 3 was an eight-day-old female Simmental calf (Fig. 1**h**-**k**). On clinical examination, the calf presented with a reduced nutritional status. Similar to case 2, it had a predominantly white coat color with two clearly visible red pigmented spots. One was triangular and located at the apex of the left ear and the other was round with a diameter of approximately three centimeters between the ears. The oculoconjuctival mucosa and muzzle were pink, the iris and sclera were pigmented, and the cilia were dyspigmented. The calf presented with diarrhea and acute bronchopneumonia. A reversible umbilical hernia of 5 cm in diameter was also noted. According to the owner, at two months of age, the animal showed retarded growth, chronic pneumonia, and worsening of the umbilical hernia and was therefore slaughtered. The clinical findings in case 3 suggested a multisystem depigmentation syndrome.

### Exclusion of dominant de novo mutations

WGS data were filtered for heterozygous variants present in each case individually and absent in controls, under the assumption that a dominant acting *de novo* mutation was the cause. Filtering for heterozygous variants present only in the genomes of individual cases revealed no evidence of post-zygotic *de novo* coding mutations private to the cases and absent from the controls including their parents (Online Resource 3). Additionally, filtering for heterozygous variants present in the genomes of the individual cases and their sires revealed no evidence of dominant *de novo* mutations private to the cases their mosaic sires and absent from the controls used (Online Resource 3). These results suggest that dominant *de novo* mutations are unlikely to explain the studied phenotypes.

### Pedigree analysis and exclusion of a common recessive allele

**Fig. 2:**
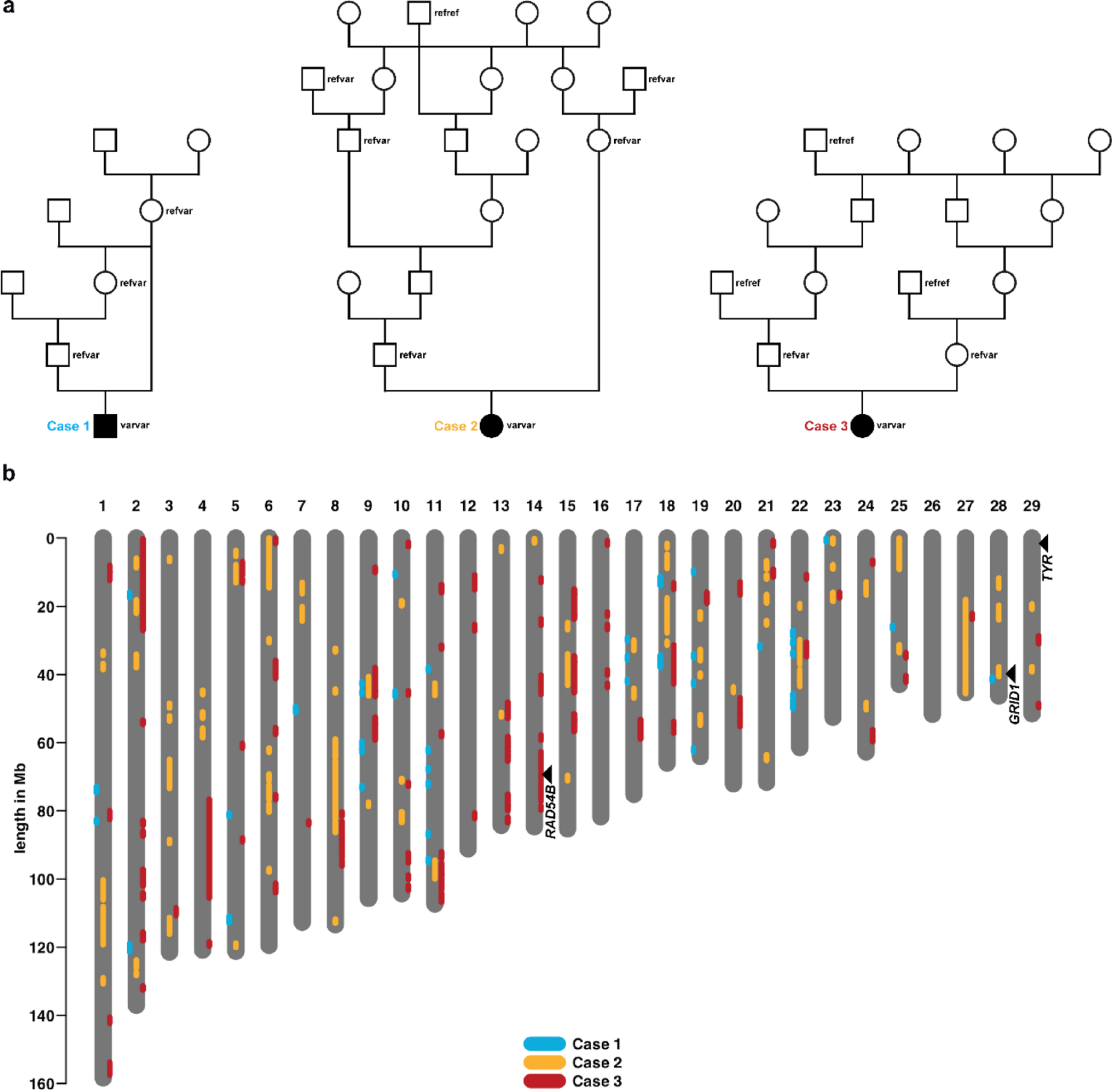
Pedigree and runs of homozygosity analyses.** a** The family history of case 1 (left), case 2 (middle) and case 3 (right) each indicates recessive inheritance. The genotypes of the identified candidate causal variants are shown below the symbols for those family members with available DNA samples. Note that, in case 2, the two male heterozygous carriers of the candidate causal variant at the top of the pedigree share two common ancestors several generations ago (not shown). **b** Runs of homozygosity larger than 1 Mb present in case 1 (blue), case 2 (orange), and case 3 (red). The black arrows indicate the location of the three genes in which candidate causal variants were identified

Pedigree records did not allow the identification of an obvious common ancestor for the three apparently unrelated cases. However, pedigree analysis of the individual cases suggested a recessive mode of inheritance, either through consanguineous mating (case 1) or inbreeding loops between the parents (cases 2 and 3; Fig. 2a).

The parentage was verified for all trios, and the mean genomic inbreeding coefficient (F_ROH) was estimated based on the ROH for all available Simmental genomes (*n* = 110). F_ROH of the three affected calves was 0.23 (case 1), 0.23 (case 2), and 0.25 (case 3), which was within the normal breed range compared to the studied population control cohort (mean 0.22, SD ± 0.04, range 0.08–0.33; Online Resource 4).

As the animals were reported to be born breed-atypically white, we grouped them together in an initial attempt to establish whether a single rare allele was responsible. No region of homozygosity shared by all three Simmental cases and absent from the parental genomes was detected (Fig. 2b; Online Resource 4). These results suggested that simple recessive inheritance due to a single common causal allele was unlikely to explain the observed congenital phenotypes in the three animals. In addition, the WGS data were filtered for homozygous coding variants that were privately present in the three Simmental calves, but no SNV or small indel variants common to all cases were identified.

### Evidence for different recessive candidate causal variants

Considering the different clinical findings, we subsequently analyzed each case separately and found several ROH of different sizes, ranging from kilobases to megabases, in each case (Online Resource 4). The average length of ROH was 388 kb for case 1, 621 kb for case 2, and 616 kb for case 3. Subsequently, the WGS data of the three cases were filtered for homozygous protein-changing variants that were individually present and rarely homozygous in controls, under the assumption that recessively inherited coding alleles cause the presented disorders. All parents were considered to be obligate heterozygous carriers. This allowed the identification of private homozygous variants affecting possible candidate genes for each case. Frequency of these SNVs was further evaluated in the global cohort of > 5000 cattle genomes (Table 1.). Read depth analysis and visual inspection of the WGS data for possible larger structural variants in the regions of the considered candidate genes revealed no evidence of such variants, either homozygous or heterozygous. The known coat color dilution-related *PMEL* variant (OMIA Variant ID 484) was present in heterozygous state in all three cases. Among parents all the three possible *PMEL* genotypes were observed (Online Resource 6).

**Table 1.** Variant classification and occurrence of the identified protein-changing variants

Case ID	Gene	Variant classification*	Breed	Global Cattle Genome Cohort**			Swiss Genotyping Cohort***			Other breeds with the variant allele detected
				var/var	ref/var	ref/ref	var/var	ref/var	ref/ref	
1	*TYR*	likely pathogenic	Simmental	1^#^	2	237	0	0	1243	
			others	0	0	5433	0	0	16,534	-
	*PTBP2*	uncertain significance	Simmental	1^#^	4	235	-	-	-	
			others	0	4	5429	-	-	-	Altai, Jersey
	*LOC787891*	likely benign	Simmental	1^#^	21	218	-	-	-	
			others	0	4	5429	-	-	-	Belgian Red White Campine, Brown Swiss
	*ZNF226*	uncertain significance	Simmental	1^#^	15	226	1	77	1333	
			others	0	4	5429	0	30	17,082	Holstein, Montbeliarde, Swiss Fleckvieh
	*FASN*	likely benign	Simmental	1^#^	11	228	20	904	9645	
			others	0	1	5432	2	231	137,487	Swiss Fleckvieh, Holstein, Busa
	*MAGI1*	uncertain significance	Simmental	1^#^	10	229	0	93	1274	
			others	0	0	5433	0	9	17,097	Swiss Fleckvieh, Holstein, Brown Swiss
2	*GRID1*	likely pathogenic	Simmental	1^#^	8	231	0	22	594	
			others	0	0	5433	0	0	1744	-
	*GRIN2A*	uncertain significance	Simmental	1^#^	7	232	20	737	9791	
			others	0	9	5424	0	72	137,655	Belgian Red White Campine, Brown Swiss, Charolais, Holstein, Marchigiana, Swiss Fleckvieh
	*OTOGL*	likely benign	Simmental	1^#^	19	220	7	237	1144	
			others	0	3	5430	0	43	16,884	Gelbvieh, Menggu, Swiss Fleckvieh, Holstein, Brown Swiss, Original Braunvieh
3	*RAD54B*	uncertain significance	Simmental	1^#^	11	228	-	-	-	
			others	1^b^	3	5429	-	-	-	Ottonese, Montbeliarde
	*NSMF*	likely benign	Simmental	1^#^	5	234	3	95	1311	
			others	0	1	5432	0	65	17,030	Swiss Fleckvieh, Holstein, Brown Swiss, Original Braunvieh

* Variant pathogenicity classification based on Boeykens et al. 2024 (for details see Online Resource 1);

** Swiss Comparative Bovine Resequencing Project and 1000 Bull Genome Project;

*** Swiss Axiom custom array genotyping data;

^**#**^ case;

^b^ Ottonese breed, unknown phenotype

**Fig. 3:**
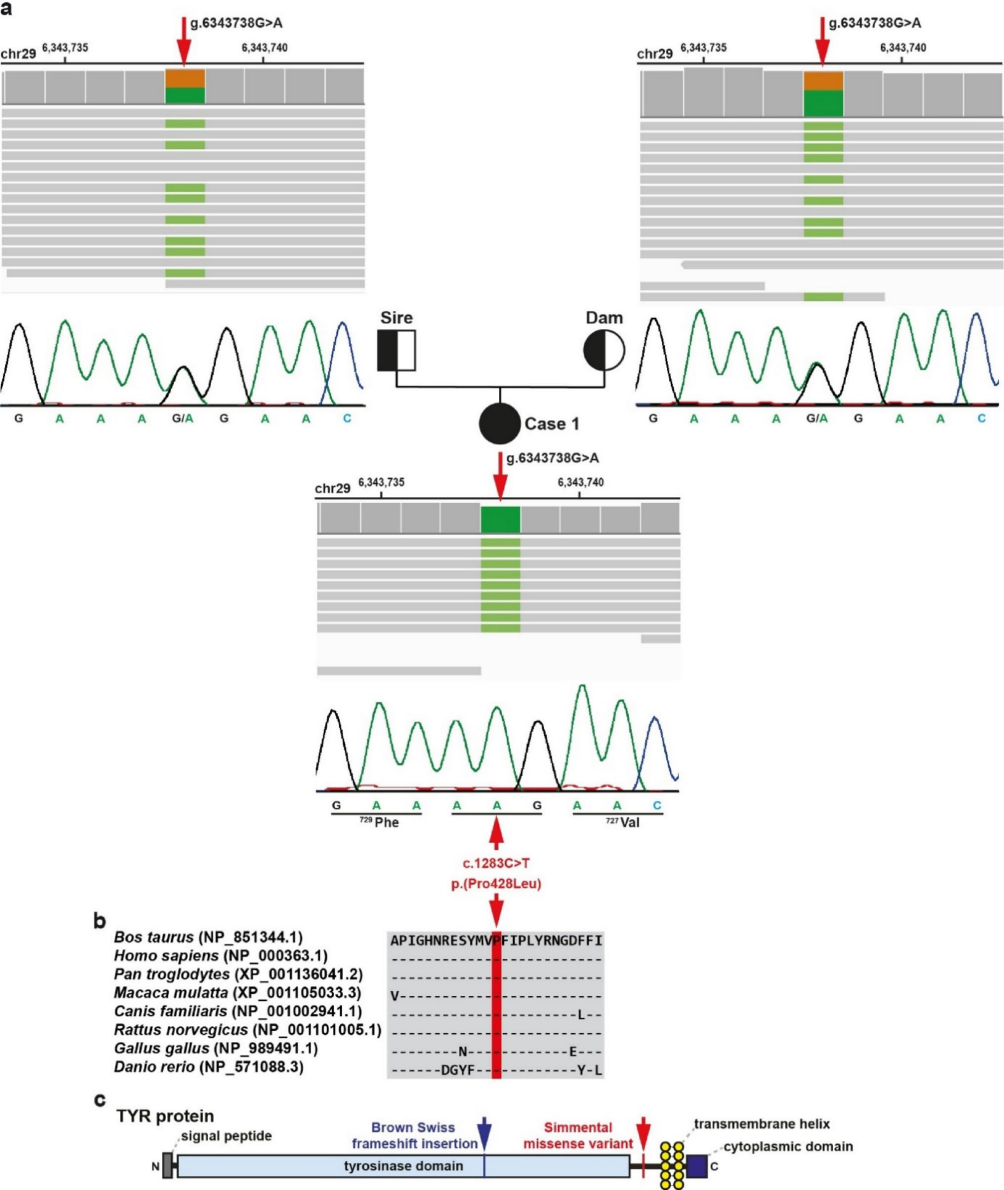
A homozygous ***TYR*** missense variant in a Simmental calf with oculocutaneous albinism type 1B.** a** IGV screenshots showing the g.6343738G > A variant on chromosome 29, homozygous in case 1 (shown below) and heterozygous in both parents (top left: sire; top right: dam) as detected by WGS. Below the IGV screenshots are the electropherograms of the parents and case obtained by Sanger sequencing. **b** Multiple sequence alignment of the TYR protein encompassing the region of the p.Pro428Leu variant showing complete evolutionary conservation. **c** Schematic representation of the bovine TYR protein and its functional domains. The blue arrow indicates the previously identified frameshift variant associated with coat/skin color, oculocutaneous albinism type I in Brown Swiss (OMIA:000202–9913). The red arrow points to the C-terminal variant identified in case 1

In case 1, variant filtering identified six homozygous SNVs with a predicted moderate impact (Table 1., Online Resource 1). Of these six, only one affected a clear candidate gene for the observed oculocutaneous albinism phenotype. This was a homozygous SNV in *TYR* exon 4 (chr29:g.6343738G > A; NM_181001.3:c.1283 C > T) and was confirmed by visual inspection and subsequent Sanger sequencing (Fig. 3a). This variant is located in a 243 kb-sized ROH on chromosome 29 (Online Resource 5). The likely pathogenic *TYR* missense variant was predicted to change the encoded amino acid of TYR residue 428 (NP_851344.1:p.Pro428Leu), which is located in the Di-copper center-containing domain (Fig. 3b, c). These molecular findings led to a precise diagnosis of oculocutaneous albinism type 1 (OCA1) in this case.

**Fig. 4:**
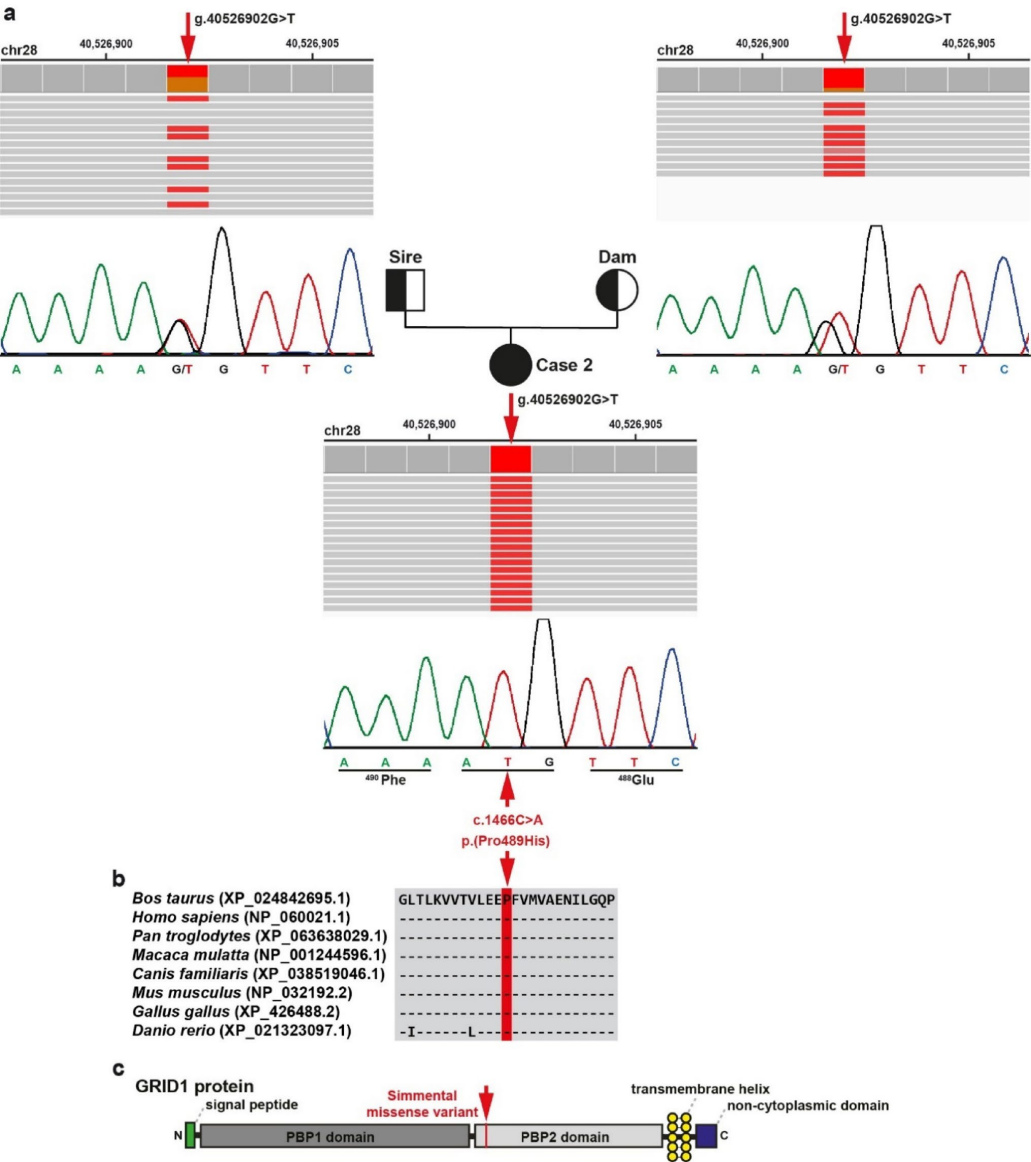
A homozygous ***GRID1*** missense variant in a Simmental calf affected by multisystem depigmentation syndrome.** a** IGV screenshots showing the g. 40526902G > T variant on chromosome 28 homozygous in case 2 (shown below) and heterozygous in both parents (top left: sire; top right: dam) as detected by WGS. Below the IGV screenshots are the electropherograms of the parents and the case obtained by Sanger sequencing. **b** Multiple sequence alignment of GRID1 protein encompassing the region of the p.Pro428Leu variant shows complete evolutionary conservation. **c** Schematic representation of the bovine GRID1 protein and its functional domains. The red arrow points to the variant identified in case 2, which is located in the ligand-binding domain of an orphan ionotropic glutamate receptor delta-1, a member of the type 2 periplasmic-binding fold protein superfamily (PBP2) domain

In case 2, filtering the WGS data revealed three homozygous SNVs with a predicted moderate impact (Table 1., Online Resource 1). Of these three, only one variant involved a potential candidate gene for the observed syndromic condition. This was a homozygous SNV in *GRID1* exon 11 (chr28:g.40526902G > T; XM_024986926.1:c.1466 C > A) that was later confirmed visually and experimentally (Fig. 4a). This variant is located in a 2.8 Mb-sized ROH on chromosome 28 (Fig. 2, Online Resource 5). The likely pathogenic *GRID1* missense variant was predicted to alter the encoded amino acid of GRID1 residue 489 (XP_024842694.1:p.Pro489His), which is included in the ligand-binding domain of an orphan ionotropic glutamate receptor delta-1, a member of the type 2 periplasmic-binding fold protein superfamily domain (Fig. 4b, c).

**Fig. 5:**
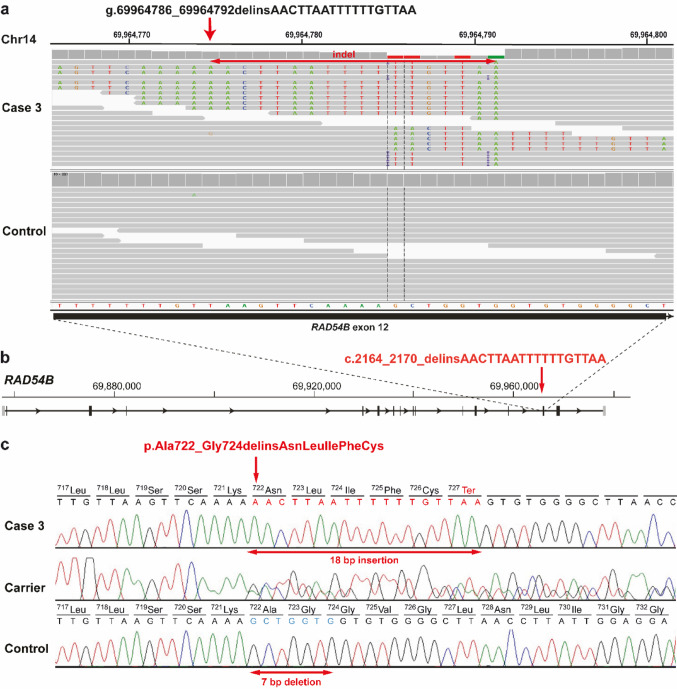
A homozygous ***RAD54B*** frameshift variant in a Simmental calf affected by short stature-auditory depigmentation syndrome.** a** IGV screenshots showing the g.69964786_69964792delinsAACTTAATTTTTTGTTAA variant on chromosome 14 homozygous in case 2 compared to a control. **b** Gene structure showing the location of the variant in *RAD54B* exon 12 (red arrow). **c** Electropherograms showing the case (var/var), a carrier (ref/var), and a control (ref/ref) genotypes obtained by Sanger sequencing. The red arrows indicate the details of the indel variant identified in case 3

In case 3, filtering identified two homozygous SNVs with a predicted high or moderate impact (Table 1., Online Resource 1). Of these two, only one variant affected a potential candidate gene for the syndromic form of depigmentation. This was a homozygous small indel variant in *RAD54B* exon 12 (chr14:g.69964786_69964792delinsAACTTAATTTTTTGTTAA; NM_001192955.1:c.2164_2170_delins) and was confirmed visually using IGV software and experimentally by PCR and Sanger sequencing (Fig. 5a, b,c). This variant is located in a 7.6 Mb-sized ROH on chromosome 14 (Fig. 2, Online Resource 5). The *RAD54B* variant of uncertain significance was predicted to cause a frameshift, resulting in the introduction of a premature stop codon (NP_001179884.1:p.Ala722_Gly724delinsAsnLeuIlePheCys*).

A detailed list of the eleven homozygous private protein-changing variants detected in all three cases and their predicted effects based on in silico tools and their evidence-based classification of the predicted pathogenicity is shown in Online Resource 2. Considering all the analyzed parameters, two out of the eleven could be classified as likely pathogenic, including the *TYR* and *GRID1* variants found in cases 1 and 2. Additionally, three variants in *MAGI1*, *PTBP2*, and *ZNF226*, which were found in case 1, were classified as of uncertain significance. Meanwhile, two variants in *FASN* and *LOC787891* were considered to be likely benign. In case 2, besides the likely pathogenic *GRID1* variant, one variant in *GRIN2A* was of uncertain significance, and one in *OTOGL* was classified as likely benign. In case 3, the frameshift variant in *RAD54B* was deemed of uncertain significance while the second variant in *NSMF* was classified as likely benign.

For eight out of the eleven homozygous protein-changing SNVs found in all three cases, additional array genotyping data were available from Swiss dairy cattle populations. These variants mostly or exclusively appeared in Simmental cattle at low allele frequencies (Table 1.). Of the six variants found in case 1, the variant in *TYR* was not detected in more than 1200 Simmental and was absent from other breeds, while there were carriers for three other variants, including homozygotes for the SNVs in *FASN* and *ZNF226*. All three variants identified in case 2 were genotyped, with no additional homozygotes observed for any of them. Only the *GRID1* variant was unique to the Simmental breed. Of the two variants found in case 3, additional genotypes were available only for the SNV in *NSMF*, showing three homozygous animals in normal-colored purebred Simmental cattle.

## Discussion

This study broadens the range of known pigmentation genes and highlights the value of domestic animals as biomedical models. We describe the clinical phenotypes of three Simmental calves with different syndromic forms of albinism and depigmentation, and we identified three rare, different recessive alleles in different genes. The unexpected genetic diversity reveals two new potential genes that might be associated with melanocytes and development. We hypothesized that the three conditions observed to be related to white coat color and diagnosed as OCA, short stature-auditory depigmentation syndrome, and multisystem depigmentation syndrome were due to altered embryonic development of the neural crest melanocyte lineage and a lack of mature melanocytes in unpigmented areas of the skin (‘leucism’). Based on Mendelian inheritance and parallels with known (de)pigmentation disorders in livestock and humans (Schmutz et al. 2004; Baxter and Pavan 2013; Rothammer et al. 2017), we conducted a genome-wide search for causal variants using a WGS trio-based approach. We first prioritized larger structural as well as protein-changing variants in known depigmentation genes, followed by all protein-coding variants in other genes. Excluding the known in-frame *PMEL* deletion, our genomic analysis revealed unexpected genetic heterogeneity underlying abnormal albinism and depigmentation in Simmental cattle.

Clinical resemblances between human and animal forms of OCA and the clinical phenotype of case 1 led to the hypothesis that genetic variants in candidate genes for OCA could be responsible for this disease in the studied Simmental calf. We identified a predicted deleterious missense variant affecting *TYR*, a plausible candidate gene for OCA, which occurred only in the pedigree of the affected animal and is apparently not widespread in the current Swiss Simmental population.

The *TYR* gene encodes the tyrosinase protein that is a copper-containing oxidase with a critical role in the formation of pigments such as melanin and other polyphenolic compounds (Lai et al. 2017). Two recessive forms of OCA (type 1 A: OCA1A; type 1B: OCA1B), associated with *TYR* have been reported in humans (OMIM:606933) (Giebel et al. 1991; Fukai et al. 1995). In this species, OCA1A and OCA1B are characterized by congenital hypopigmentation of the skin, hair, and eyes, as well as ocular abnormalities (King et al. 2003). However, they differ significantly in terms of the severity and progression of pigmentation (Grønskov et al. 2007). OCA1A is caused by variants that result in loss-of-function of tyrosinase activity (Patel and Sergeev 2020). As a result, individuals with OCA1A do not produce melanin and therefore have congenital white hair, very pale skin, light blue irises that remain unchanged throughout life, and severe eye abnormalities (Grønskov et al. 2007). In contrast, OCA1B results from hypomorphic variants that allow for residual tyrosinase activity, resulting in some melanin production (Patel and Sergeev 2020). Individuals are typically born with minimal pigmentation, but can accumulate more pigment in their skin, hair, and eyes over time. Hair color may change from white or yellow to blonde or light brown, and the skin may develop a slight tan. In addition, eye abnormalities such as visual impairment are less severe than in OCA1A (Patel and Sergeev 2020). The OCA-affected Simmental calf had markedly reduced pigmentation, ocular photosensitivity, partially pigmented claws and carried a homozygous missense variant (p.Pro428Leu) in an evolutionary conserved domain of the TYR protein. We speculate that this variant impairs but does not abolish tyrosinase activity. Given the phenotypic and genotypic similarities to human patients with OCA1B, the calf was finally diagnosed with OCA1B. In cattle, only one case of *TYR*-related form of OCA1A has previously been reported in a single Braunvieh calf (Schmutz et al. 2004). Therefore, we propose the *TYR* variant as a candidate causal for the observed phenotype. Given the obvious consanguineous mating, we expected the affected animal to exhibit higher genomic inbreeding and a longer ROH than the observed 243 kb-sized genome segment in that the *TYR* variant is located. However, given the absence of the variant allele in the Simmental population, we propose that the mutation in the *TYR* gene probably occurred recently within the maternal lineage of the dam, resulting in a shortened haplotype due to recombination, and is therefore limited to that specific cattle family.

In the other two Simmental cases with syndromic white coat color phenotypes, no protein-changing variants were detected in previously known depigmentation candidate genes. 2 had a predominantly white coat with isolated areas of residual pigmentation, proportionate short stature, brachygnathia, and reduced responsiveness to auditory stimuli, features consistent with a short stature–auditory depigmentation syndrome. Filtering for rare protein-changing variants in this case identified a homozygous missense variant in *GRID1*. The p.Pro489His variant affects a conserved amino acid and is predicted to be deleterious by several in silico tools, suggesting a likely effect on protein function. Importantly, this variant was only observed in a homozygous state in the affected calf, showing breed specificity as it was absent from more than 5,400 control genomes representing different cattle breeds. Within the Simmental breed, it had a low allele frequency (0.0187) with no additional homozygous individuals detected. The nature of this variant, combined with its predicted functional impact and exclusive homozygosity in the affected animal, supports the *GRID1* missense variant as a candidate causal for the observed phenotype. It represents a rare deleterious allele that is limited to Swiss Simmental cattle, and the length of the associated haplotype (2.8 Mb) suggests that the mutation occurred more recently.

*GRID1* encodes the glutamate receptor ionotropic delta-1 protein, which plays a critical role in synaptic organization and signaling in the central nervous system (Piot et al. 2023). Notably, *GRID1* is also moderately expressed in human skin and melanocytes (Uhlén et al. 2015; Bastian et al. 2025), suggesting potential relevance beyond neural tissues. *GRID1* is predicted to interact with 19 genes (Oughtred et al. 2021), including *PDGFRA*. *PDGFRA* is a well-established regulator of melanocyte development and coat color variation in several species, including pigs, goats, cattle, and sheep (Hanna et al. 2014; Nazari-Ghadikolaei et al. 2018; Baazaoui et al. 2019; Xu et al. 2024). Disruptions in this gene can impair melanocyte migration, leading to hypopigmentation. For example, in Wuzhishan pigs, *PDGFRA* is significantly upregulated in pigmented skin compared to non-pigmented regions, suggesting a role in melanin deposition (Xu et al. 2024). Furthermore, *PDGFRA* often co-localizes with *KIT*, a pigmentation-associated gene, acting synergistically to modulate melanocyte survival and differentiation (Hanna et al. 2014; Nazari-Ghadikolaei et al. 2018; Baazaoui et al. 2019). Taken together, the gene function, interactions, and functional annotations suggest a broader biological role for *GRID1* in pathways relevant to development and pigmentation syndromes. The identified *GRID1* missense variant, although not yet characterized in functional studies, may disrupt normal molecular interactions with, for example *PDGFRA*, leading to aberrant signaling during development. Such dysregulation could affect melanin deposition in the skin, resulting in hypopigmentation.

In case 3, the prioritization and filtering criteria led to the detection of a rare homozygous frameshift variant in *RAD54B*, most likely representing a loss-of-function allele. Clinically, this calf presented with a predominantly white coat color and isolated residual pigmentation, failure to thrive, recurrent respiratory infections, and an umbilical hernia, features that together suggest a multisystem depigmentation syndrome. The identified variant in *RAD54B* is extremely rare across breeds, detected in heterozygous state only in some Simmental and Montbéliarde cattle. In addition, a single homozygous Ottonese cattle with an unknown phenotype was identified in the 1000 Bull Genomes Project variant dataset. However, due to the lack of access to the corresponding BAM files, we were unable to visually inspect the alignment using IGV to confirm the precise *RAD54B* genotype of this animal. Therefore, given the complexity of the identified indel variant, the accuracy of the reported homozygous genotype of this individual remains uncertain. The length of the associated haplotype (7.6 Mb) in case 3 suggests that the derived allele occurred recently although it seems to appear in cattle of other breeds, however, this could not be verified.

*RAD54B* encodes a DNA repair and recombination protein involved in homologous recombination, particularly in resolving of DNA double-strand breaks (Miyagawa 2002). *RAD54B* is predicted to interact with 59 genes (Oughtred et al. 2021). Interestingly, it interacts with *WRN*, *MDM2*, and *RAD51* - genes reported to be involved in syndromic phenotypes affecting pigmentation. Notably, *WRN* in humans is associated with Werner syndrome (OMIM:277700), a recessively inherited disorder characterized among other features by short stature, premature ageing, retinal degeneration, early greying of the hair, scleroderma-like skin changes, and hypogonadism (Oshima et al. 2017). Variants in *MDM2* (OMIM:618681) have been associated with Lessel–Kubisch syndrome, a recessive condition with phenotypic overlap with Werner syndrome, including short stature, craniofacial dysmorphism, hypogonadism, and premature greying (Lessel et al. 2017). In human patients, *RAD51* is associated with the dominantly inherited Fanconi anemia, complementation group R (OMIM:617244). Affected patients present with short stature, craniofacial dysmorphism, intellectual disability, abnormal skin pigmentation including hyper- or hypopigmentation, ophthalmic, and genitourinary tract anomalies (Ameziane et al. 2015; Mehta and Ebens 2021). Taken together, these gene interactions and the overlapping human syndromic phenotypes support a plausible role for *RAD54B* in regulating developmental processes such as growth and pigmentation. The frameshift variant identified in the affected Simmental calf is predicted to cause premature truncation of the *RAD54B* protein if expressed, which would likely abolish its role in DNA double-strand break repair. Such loss-of-function allele could affect genome integrity in rapidly dividing or differentiation-sensitive cell populations, including melanocytes, and growth-regulating tissues. Given the interaction of *RAD54B* with known disease-associated genes such as *WRN*, *MDM2*, and *RAD51*, its disruption may trigger downstream effects that resemble human syndromes associated with DNA repair abnormalities. While further functional validation is required, these findings suggest that *RAD54B* may represent a previously unrecognized contributor to syndromic forms of developmental disorders with depigmentation in mammals.

The latter two cases highlight potential new candidate genes, *GRID1* and *RAD54B*, and a possible convergence of pathways underlying syndromic developmental and pigmentation disorders in mammals. Further experimental studies are required to elucidate the molecular consequences of the identified variants at the protein level and within cellular pathways, which could present a challenge given the developmental nature of the conditions. In addition, expanding this research to include more cases of identical or similar phenotypes in cattle, as well as in other animals or even other mammalian species, would provide further support for our hypothesis that variants in *GRID1* and *RAD54B* are associated with syndromic depigmentation phenotypes. Therefore, we recommend ongoing monitoring of the three proposed candidate causal variants in cattle by incorporating them into routine genotyping, in order to identify further homozygotes and add these genes to the panel of candidate genes in human genetics.

Several methodological limitations are acknowledged, including the incomplete annotation of the bovine genome, the targeted focus on larger structural variants and coding variants within candidate genes selected through literature-based approaches, and the omission of intermediate-sized structural variants, and the non-survey for intermediate sized structural variants. Additional limitations arise from the use of short-read WGS technology, which may introduce inaccuracies in read alignment and variant calling (Caspar et al. 2018), as well as from the potential influence of epigenetics or non-genetic factors.

Our study identified three different and rare recessive alleles likely associated with syndromic forms of albinism and depigmentation in Simmental cattle, affecting *TYR*, *GRID1*, and *RAD54B*. These findings reveal unexpected genetic heterogeneity underlying pigment disorders in livestock and introduce two potential novel candidate genes (*GRID1* and *RAD54B*) with currently unknown roles in melanocyte development and syndromic conditions. Therefore, this study possibly expands the spectrum of pigmentation-related genes in mammals and highlights the value of studying naturally occurring phenotypes in domestic animals as biomedical models. It is important to note that further functional validation and case collection are required to confirm the pathogenicity of these variants and to explore their broader relevance across species. These findings lay the groundwork for enhanced monitoring of the identified variants and potential genetic diagnostics in cattle, and they may offer a basis for future research on pigmentation in mammals.

## Conclusions

Rare non-lethal conditions like syndromic forms of white coat color in Simmental cattle, are often under-reported or misdiagnosed in livestock, even though they can have a detrimental impact on the quality of life of the affected animals. This study demonstrates how combining clinical phenotyping and genomic data from farm animals can be used to identify novel genetic variants for Mendelian disorders. This study emphasizes the value of precision diagnostics in understanding rare disorders, and the importance of monitoring cattle breeding populations for harmful genetic variants. The results obtained in this study have two main implications. Firstly, they enable breeders to select against the deleterious *TYR* allele. Secondly, they possibly expand the range of depigmentation-related genes to include *GRID1* and *RAD54B* in mammals. This demonstrates the biomedical potential of studying rare congenital conditions in cattle.

## Supplementary Information

Below is the link to the electronic supplementary material.

Supplementary Material 1

Supplementary Material 2

Supplementary Material 3

Supplementary Material 4

Supplementary Material 5

Supplementary Material 6

## Supplementary Information

Below is the link to the electronic supplementary material.

## Data Availability

The whole-genome sequencing data has been made freely available under study accession numbers PRJEB18113 and PRJEB83441 in the European Nucleotide Archive (http://www.ebi.ac.uk/ena). All individual accession numbers of the WGS are available in Online Resource 1.
