# Supplementary figures and images for: Rare phenotypes of white coat color in Simmental calves: genetic causes of syndromic forms of albinism and depigmentation
Source: Mol Genet Genomics. 2025 Sep 6;300(1):91. doi: 10.1007/s00438-025-02290-2 (PMC12414089; doi:10.1007/s00438-025-02290-2)

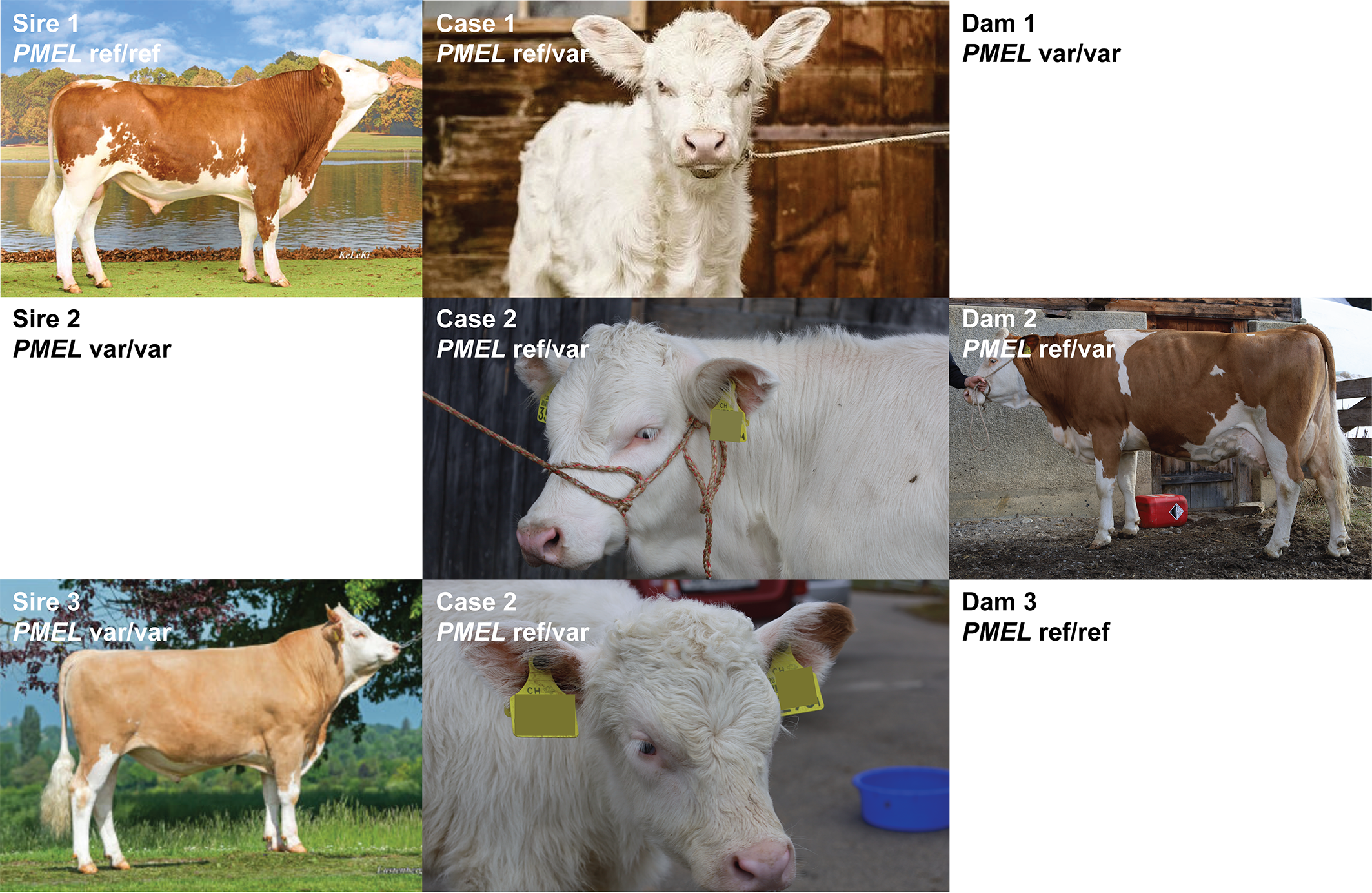

Supplement: Supplementary file 6 — Supplementary Material 6 [file 438_2025_2290_MOESM6_ESM.tif]
